# The role of artificial intelligence in achieving the Sustainable Development Goals

**DOI:** 10.1038/s41467-019-14108-y

**Published:** 2020-01-13

**Authors:** Ricardo Vinuesa, Hossein Azizpour, Iolanda Leite, Madeline Balaam, Virginia Dignum, Sami Domisch, Anna Felländer, Simone Daniela Langhans, Max Tegmark, Francesco Fuso Nerini

**Affiliations:** 1 Linné FLOW Centre, KTH Mechanics, SE-100 44 Stockholm, Sweden; 2 Division of Robotics, Perception, and Learning, School of EECS, KTH Royal Institute Of Technology, Stockholm, Sweden; 3 Division of Media Technology and Interaction Design, KTH Royal Institute of Technology, Lindstedtsvägen 3, Stockholm, Sweden; 4 Responsible AI Group, Department of Computing Sciences, Umeå University, SE-90358 Umeå, Sweden; 5 Leibniz-Institute of Freshwater Ecology and Inland Fisheries, Müggelseedamm 310, 12587 Berlin, Germany; 6 AI Sustainability Center, SE-114 34 Stockholm, Sweden; 7 Basque Centre for Climate Change (BC3), 48940 Leioa, Spain; 8 Department of Zoology, University of Otago, 340 Great King Street, 9016 Dunedin, New Zealand; 9 Center for Brains, Minds and Machines, Massachusetts Institute of Technology, Cambridge, Massachusetts 02139 USA; 10 Unit of Energy Systems Analysis (dESA), KTH Royal Institute of Technology, Brinellvagen, 68SE-1004 Stockholm, Sweden

**Keywords:** Computational science, Developing world, Energy efficiency

## Abstract

The emergence of artificial intelligence (AI) and its progressively wider impact on many sectors requires an assessment of its effect on the achievement of the Sustainable Development Goals. Using a consensus-based expert elicitation process, we find that AI can enable the accomplishment of 134 targets across all the goals, but it may also inhibit 59 targets. However, current research foci overlook important aspects. The fast development of AI needs to be supported by the necessary regulatory insight and oversight for AI-based technologies to enable sustainable development. Failure to do so could result in gaps in transparency, safety, and ethical standards.

## Abstract (web summary)

Artificial intelligence (AI) is becoming more and more common in people’s lives. Here, the authors use an expert elicitation method to understand how AI may affect the achievement of the Sustainable Development Goals.

## Introduction

The emergence of artificial intelligence (AI) is shaping an increasing range of sectors. For instance, AI is expected to affect global productivity^1^, equality and inclusion^2^, environmental outcomes^3^, and several other areas, both in the short and long term^4^. Reported potential impacts of AI indicate both positive^5^ and negative^6^ impacts on sustainable development. However, to date, there is no published study systematically assessing the extent to which AI might impact all aspects of sustainable development—defined in this study as the 17 Sustainable Development Goals (SDGs) and 169 targets internationally agreed in the 2030 Agenda for Sustainable Development^7^. This is a critical research gap, as we find that AI may influence the ability to meet all SDGs.

**Fig. 1:**
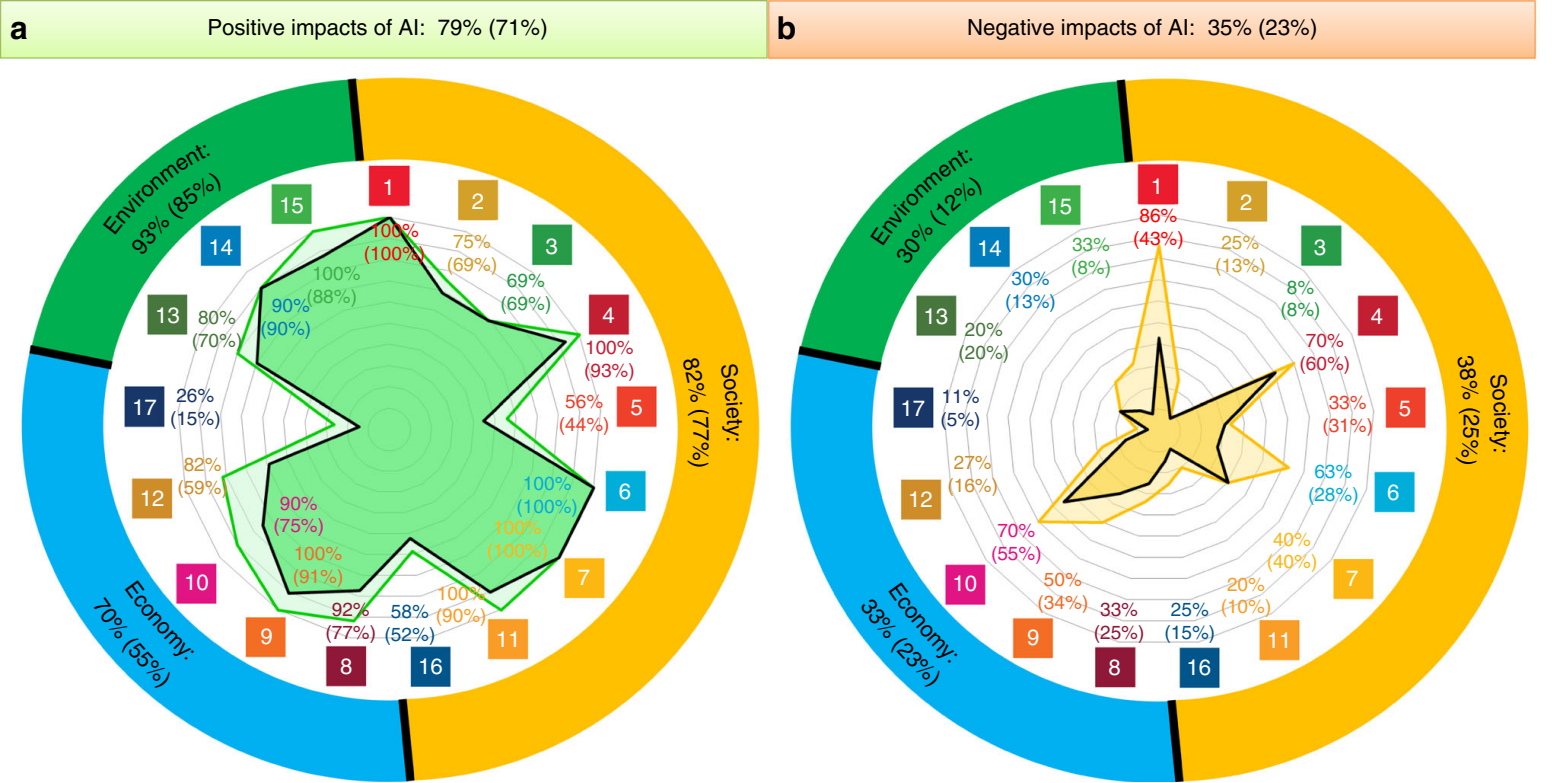
Summary of positive and negative impact of AI on the various SDGs. Documented evidence of the potential of AI acting as (**a**) an enabler or (**b**) an inhibitor on each of the SDGs. The numbers inside the colored squares represent each of the SDGs (see the Supplementary Data 1). The percentages on the top indicate the proportion of all targets potentially affected by AI and the ones in the inner circle of the figure correspond to proportions within each SDG. The results corresponding to the three main groups, namely Society, Economy, and Environment, are also shown in the outer circle of the figure. The results obtained when the type of evidence is taken into account are shown by the inner shaded area and the values in brackets.

Here we present and discuss implications of how AI can either enable or inhibit the delivery of all 17 goals and 169 targets recognized in the 2030 Agenda for Sustainable Development. Relationships were characterized by the methods reported at the end of this study, which can be summarized as a consensus-based expert elicitation process, informed by previous studies aimed at mapping SDGs interlinkages^8–10^. A summary of the results is given in Fig. 1 and the Supplementary Data 1 provides a complete list of all the SDGs and targets, together with the detailed results from this work. Although there is no internationally agreed definition of AI, for this study we considered as AI any software technology with at least one of the following capabilities: perception—including audio, visual, textual, and tactile (e.g., face recognition), decision-making (e.g., medical diagnosis systems), prediction (e.g., weather forecast), automatic knowledge extraction and pattern recognition from data (e.g., discovery of fake news circles in social media), interactive communication (e.g., social robots or chat bots), and logical reasoning (e.g., theory development from premises). This view encompasses a large variety of subfields, including machine learning.

## Documented connections between AI and the SDGs

Our review of relevant evidence shows that AI may act as an enabler on 134 targets (79%) across all SDGs, generally through a technological improvement, which may allow to overcome certain present limitations. However, 59 targets (35%, also across all SDGs) may experience a negative impact from the development of AI. For the purpose of this study, we divide the SDGs into three categories, according to the three pillars of sustainable development, namely Society, Economy, and Environment^11,12^ (see the Methods section). This classification allows us to provide an overview of the general areas of influence of AI. In Fig. 1, we also provide the results obtained when weighting how appropriate is the evidence presented in each reference to assess an interlinkage to the percentage of targets assessed, as discussed in the Methods section and below. A detailed assessment of the Society, Economy, and Environment groups, together with illustrative examples, are discussed next.

### AI and societal outcomes

**Fig. 2:**
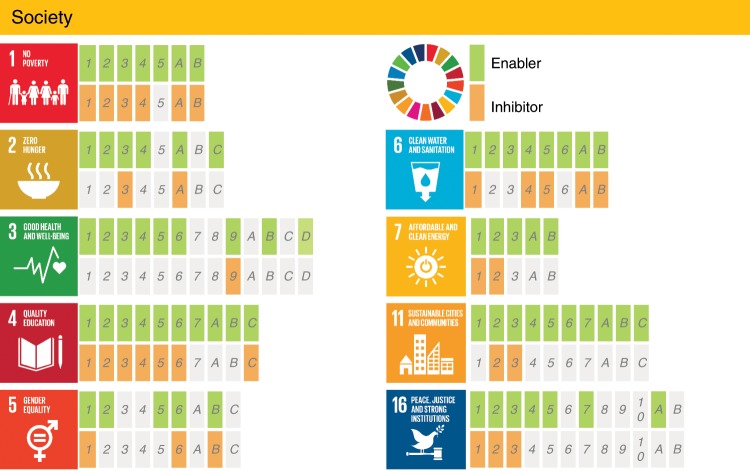
Detailed assessment of the impact of AI on the SDGs within the Society group. Documented evidence of positive or negative impact of AI on the achievement of each of the targets from SDGs 1, 2, 3, 4, 5, 6, 7, 11, and 16 (https://www.un.org/sustainabledevelopment/). Each block in the diagram represents a target (see the Supplementary Data 1 for additional details on the targets). For targets highlighted in green or orange, we found published evidence that AI could potentially enable or inhibit such target, respectively. The absence of highlighting indicates the absence of identified evidence. It is noteworthy that this does not necessarily imply the absence of a relationship. (The content of of this figure has not been reviewed by the United Nations and does not reflect its views).

Sixty-seven targets (82%) within the Society group could potentially benefit from AI-based technologies (Fig. 2). For instance, in SDG 1 on no poverty, SDG 4 on quality education, SDG 6 on clean water and sanitation, SDG 7 on affordable and clean energy, and SDG 11 on sustainable cities, AI may act as an enabler for all the targets by supporting the provision of food, health, water, and energy services to the population. It can also underpin low-carbon systems, for instance, by supporting the creation of circular economies and smart cities that efficiently use their resources^13,14^. For example, AI can enable smart and low-carbon cities encompassing a range of interconnected technologies such as electrical autonomous vehicles and smart appliances that can enable demand response in the electricity sector^13,14^ (with benefits across SDGs 7, 11, and 13 on climate action). AI can also help to integrate variable renewables by enabling smart grids that partially match electrical demand to times when the sun is shining and the wind is blowing^13^. Fewer targets in the Society group can be impacted negatively by AI (31 targets, 38%) than the ones with positive impact. However, their consideration is crucial. Many of these relate to how the technological improvements enabled by AI may be implemented in countries with different cultural values and wealth. Advanced AI technology, research, and product design may require massive computational resources only available through large computing centers. These facilities have a very high energy requirement and carbon footprint^15^. For instance, cryptocurrency applications such as Bitcoin are globally using as much electricity as some nations’ electrical demand^16^, compromising outcomes in the SDG 7 sphere, but also on SDG 13 on Climate Action. Some estimates suggest that the total electricity demand of information and communications technologies (ICTs) could require up to 20% of the global electricity demand by 2030, from around 1% today^15^. Green growth of ICT technology is therefore essential^17^. More efficient cooling systems for data centers, broader energy efficiency, and renewable-energy usage in ICTs will all play a role in containing the electricity demand growth^15^. In addition to more efficient and renewable-energy-based data centers, it is essential to embed human knowledge in the development of AI models. Besides the fact that the human brain consumes much less energy than what is used to train AI models, the available knowledge introduced in the model (see, for instance, physics-informed deep learning^18^) does not need to be learnt through data-intensive training, a fact that may significantly reduce the associated energy consumption. Although AI-enabled technology can act as a catalyst to achieve the 2030 Agenda, it may also trigger inequalities that may act as inhibitors on SDGs 1, 4, and 5. This duality is reflected in target 1.1, as AI can help to identify areas of poverty and foster international action using satellite images^5^. On the other hand, it may also lead to additional qualification requirements for any job, consequently increasing the inherent inequalities^19^ and acting as an inhibitor towards the achievement of this target.

Another important drawback of AI-based developments is that they are traditionally based on the needs and values of nations in which AI is being developed. If AI technology and big data are used in regions where ethical scrutiny, transparency, and democratic control are lacking, AI might enable nationalism, hate towards minorities, and bias election outcomes^20^. The term “big nudging” has emerged to represent using big data and AI to exploit psychological weaknesses to steer decisions—creating problems such as damaging social cohesion, democratic principles, and even human rights^21^. AI has been recently utilized to develop citizen scores, which are used to control social behavior^22^. This type of score is a clear example of threat to human rights due to AI misuse and one of its biggest problems is the lack of information received by the citizens on the type of analyzed data and the consequences this may have on their lives. It is also important to note that AI technology is unevenly distributed: for instance, complex AI-enhanced agricultural equipment may not be accessible to small farmers and thus produce an increased gap with respect to larger producers in more developed economies^23^, consequently inhibiting the achievement of some targets of SDG 2 on zero hunger. There is another important shortcoming of AI in the context of SDG 5 on gender equality: there is insufficient research assessing the potential impact of technologies such as smart algorithms, image recognition, or reinforced learning on discrimination against women and minorities. For instance, machine-learning algorithms uncritically trained on regular news articles will inadvertently learn and reproduce the societal biases against women and girls, which are embedded in current languages. Word embeddings, a popular technique in natural language processing, have been found to exacerbate existing gender stereotypes^2^. In addition to the lack of diversity in datasets, another main issue is the lack of gender, racial, and ethnic diversity in the AI workforce^24^. Diversity is one of the main principles supporting innovation and societal resilience, which will become essential in a society exposed to changes associated to AI development^25^. Societal resilience is also promoted by decentralization, i.e., by the implementation of AI technologies adapted to the cultural background and the particular needs of different regions.

### AI and economic outcomes

**Fig. 3:**
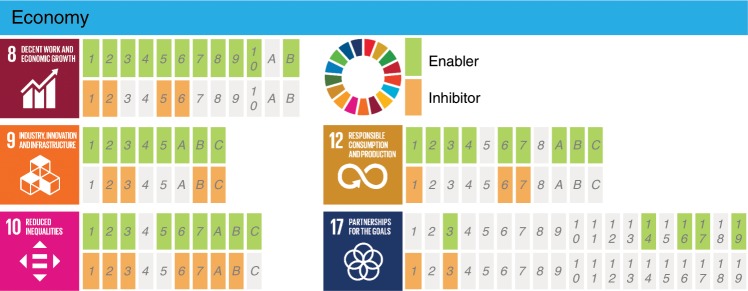
Detailed assessment of the impact of AI on the SDGs within the Economy group. Documented evidence of positive or negative impact of AI on the achievement of each of the targets from SDGs 8, 9, 10, 12, and 17 (https://www.un.org/sustainabledevelopment/). The interpretation of the blocks and colors is as in Fig. 2.  (The content of of this figure has not been reviewed by the United Nations and does not reflect its views).

The technological advantages provided by AI may also have a positive impact on the achievement of a number of SDGs within the Economy group. We have identified benefits from AI on 42 targets (70%) from these SDGs, whereas negative impacts are reported in 20 targets (33%), as shown in Fig. 1. Although Acemoglu and Restrepo^1^ report a net positive impact of AI-enabled technologies associated to increased productivity, the literature also reflects potential negative impacts mainly related to increased inequalities^26–29^. In the context of the Economy group of SDGs, if future markets rely heavily on data analysis and these resources are not equally available in low- and middle- income countries, the economical gap may be significantly increased due to the newly introduced inequalities^30,31^ significantly impacting SDGs 8 (decent work and economic growth), 9 (industry, innovation and infrastructure), and 10 (reduced inequalities). Brynjolfsson and McAfee^31^ argue that AI can exacerbate inequality also within nations. By replacing old jobs with ones requiring more skills, technology disproportionately rewards the educated: since the mid 1970s, the salaries in the United States (US) salaries rose about 25% for those with graduate degrees, while the average high-school dropout took a 30% pay cut. Moreover, automation shifts corporate income to those who own companies from those who work there. Such transfer of revenue from workers to investors helps explain why, even though the combined revenues of Detroit's “Big 3” (GM, Ford, and Chrysler) in 1990 were almost identical to those of Silicon Valley's “Big 3” (Google, Apple, and Facebook) in 2014, the latter had 9 times fewer employees and were worth 30 times more on the stock market^32^. Figure 3 shows an assessment of the documented positive and negative effects on the various targets within the SDGs in the Economy group.

Although the identified linkages in the Economy group are mainly positive, trade-offs cannot be neglected. For instance, AI can have a negative effect on social media usage, by showing users content specifically suited to their preconceived ideas. This may lead to political polarization^33^ and affect social cohesion^21^ with consequences in the context of SDG 10 on reduced inequalities. On the other hand, AI can help identify sources of inequality and conflict^34,35^, and therewith potentially reduce inequalities, for instance, by using simulations to assess how virtual societies may respond to changes. However, there is an underlying risk when using AI to evaluate and predict human behavior, which is the inherent bias in the data. It has been reported that a number of discriminatory challenges are faced in the automated targeting of online job advertising using AI^35^, essentially related to the previous biases in selection processes conducted by human recruiters. The work by Dalenberg^35^ highlights the need of modifying the data preparation process and explicitly adapting the AI-based algorithms used for selection processes to avoid such biases.

### AI and environmental outcomes

**Fig. 4:**
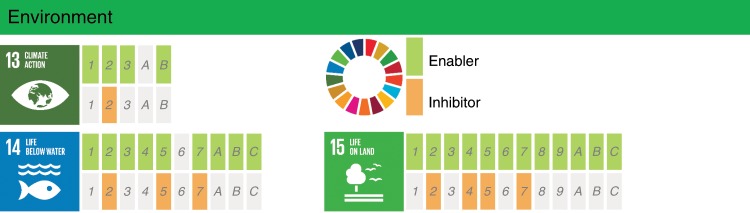
Detailed assessment of the impact of AI on the SDGs within the Environment group. Documented evidence of positive or negative impact of AI on the achievement of each of the targets from SDGs 13, 14, and 15 (https://www.un.org/sustainabledevelopment/). The interpretation of the blocks and colors is as in Fig. 2. (The content of of this figure has not been reviewed by the United Nations and does not reflect its views).

The last group of SDGs, i.e., the one related to Environment, is analyzed in Fig. 4. The three SDGs in this group are related to climate action, life below water and life on land (SDGs 13, 14, and 15). For the Environment group, we identified 25 targets (93%) for which AI could act as an enabler. Benefits from AI could be derived by the possibility of analyzing large-scale interconnected databases to develop joint actions aimed at preserving the environment. Looking at SDG 13 on climate action, there is evidence that AI advances will support the understanding of climate change and the modeling of its possible impacts. Furthermore, AI will support low-carbon energy systems with high integration of renewable energy and energy efficiency, which are all needed to address climate change^13,36,37^. AI can also be used to help improve the health of ecosystems. The achievement of target 14.1, calling to prevent and significantly reduce marine pollution of all kinds, can benefit from AI through algorithms for automatic identification of possible oil spills^38^. Another example is target 15.3, which calls for combating desertification and restoring degraded land and soil. According to Mohamadi et al.^39^, neural networks and objective-oriented techniques can be used to improve the classification of vegetation cover types based on satellite images, with the possibility of processing large amounts of images in a relatively short time. These AI techniques can help to identify desertification trends over large areas, information that is relevant for environmental planning, decision-making, and management to avoid further desertification, or help reverse trends by identifying the major drivers. However, as pointed out above, efforts to achieve SDG 13 on climate action could be undermined by the high-energy needs for AI applications, especially if non carbon-neutral energy sources are used. Furthermore, despite the many examples of how AI is increasingly applied to improve biodiversity monitoring and conservation^40^, it can be conjectured that an increased access to AI-related information of ecosystems may drive over-exploitation of resources, although such misuse has so far not been sufficiently documented. This aspect is further discussed below, where currently identified gaps in AI research are considered.

### An assessment of the collected evidence on the interlinkages

A deeper analysis of the gathered evidence was undertaken as shown in Fig. 1 (and explained in the Methods section). In practice, each interlinkage was weighted based on the applicability and appropriateness of each of the references to assess a specific interlinkage—and possibly identify research gaps. Although accounting for the type of evidence has a relatively small effect on the positive impacts (we see a reduction of positively affected targets from 79% to 71%), we observe a more significant reduction (from 35% to 23%) in the targets with negative impact of AI. This can be partly due the fact that AI research typically involves quantitative methods that would bias the results towards the positive effects. However, there are some differences across the Society, Economy and Environment spheres. In the Society sphere, when weighting the appropriateness of evidence, positively affected targets diminish by 5 percentage points (p.p.) and negatively affected targets by 13 p.p. In particular, weighting the appropriateness of evidence on negative impacts on SDG 1 (on no poverty) and SDG 6 (on clean water and sanitation) reduces the fraction of affected targets by 43 p.p. and 35 p.p., respectively. In the Economy group instead, positive impacts are reduced more (15 p.p.) than negative ones (10 p.p.) when taking into account the appropriateness of the found evidence to speak of the issues. This can be related to the extensive study in literature assessing the displacement of jobs due to AI (because of clear policy and societal concerns), but overall the longer-term benefits of AI on the economy are perhaps not so extensively characterized by currently available methods. Finally, although the weighting of evidence decreases the positive impacts of AI on the Environment group only by 8 p.p., the negative impacts see the largest average reduction (18 p.p.). This is explained by the fact that, although there are some indications of the potential negative impact of AI on this SDG, there is no strong evidence (in any of the targets) supporting this claim, and therefore this is a relevant area for future research.

In general, the fact that the evidence on interlinkages between AI and the large majority of targets is not based on tailored analyses and tools to refer to that particular issue provides a strong rationale to address a number of research gaps, which are identified and listed in the section below.

## Research gaps on the role of AI in sustainable development

The more we enable SDGs by deploying AI applications, from autonomous vehicles^41^ to AI-powered healthcare solutions^42^ and smart electrical grids^13^, the more important it becomes to invest in the AI safety research needed to keep these systems robust and beneficial, so as to prevent them from malfunctioning, or from getting hacked^43^. A crucial research venue for a safe integration of AI is understanding catastrophes, which can be enabled by a systemic fault in AI technology. For instance, a recent World Economic Forum (WEF) report raises such a concern due to the integration of AI in the financial sector^44^. It is therefore very important to raise awareness on the risks associated to possible failures of AI systems in a society progressively more dependent on this technology. Furthermore, although we were able to find numerous studies suggesting that AI can potentially serve as an enabler for many SDG targets and indicators, a significant fraction of these studies have been conducted in controlled laboratory environments, based on limited datasets or using prototypes^45–47^. Hence, extrapolating this information to evaluate the real-world effects often remains a challenge. This is particularly true when measuring the impact of AI across broader scales, both temporally and spatially. We acknowledge that conducting controlled experimental trials for evaluating real-world impacts of AI can result in depicting a snapshot situation, where AI tools are tailored towards that specific environment. However, as society is constantly changing (also due to factors including non-AI-based technological advances), the requirements set for AI are changing as well, resulting in a feedback loop with interactions between society and AI. Another underemphasized aspect in existing literature is the resilience of the society towards AI-enabled changes. Therefore, novel methodologies are required to ensure that the impact of new technologies are assessed from the points of view of efficiency, ethics, and sustainability, prior to launching large-scale AI deployments. In this sense, research aimed at obtaining insight on the reasons for failure of AI systems, introducing combined human–machine analysis tools^48^, are an essential step towards accountable AI technology, given the large risk associated to such a failure.

Although we found more published evidence of AI serving as an enabler than as an inhibitor on the SDGs, there are at least two important aspects that should be considered. First, self-interest can be expected to bias the AI research community and industry towards publishing positive results. Second, discovering detrimental aspects of AI may require longer-term studies and, as mentioned above, there are not many established evaluation methodologies available to do so. Bias towards publishing positive results is particularly apparent in the SDGs corresponding to the Environment group. A good example of this bias is target 14.5 on conserving coastal and marine areas, where machine-learning algorithms can provide optimum solutions given a wide range of parameters regarding the best choice of areas to include in conservation networks^49^. However, even if the solutions are optimal from a mathematical point of view (given a certain range of selected parameters), additional research would be needed to assess the long-term impact of such algorithms on equity and fairness^6^, precisely because of the unknown factors that may come into play. Regarding the second point stated above, it is likely that the AI projects with the highest potential of maximizing profit will get funded. Without control, research on AI is expected to be directed towards AI applications where funding and commercial interests are. This may result in increased inequality^50^. Consequently, there is the risk that AI-based technologies with potential to achieve certain SDGs may not be prioritized, if their expected economic impact is not high. Furthermore, it is essential to promote the development of initiatives to assess the societal, ethical, legal, and environmental implications of new AI technologies.

Substantive research and application of AI technologies to SDGs is concerned with the development of better data-mining and machine-learning techniques for the prediction of certain events. This is the case of applications such as forecasting extreme weather conditions or predicting recidivist offender behavior. The expectation with this research is to allow the preparation and response for a wide range of events. However, there is a research gap in real-world applications of such systems, e.g., by governments (as discussed above). Institutions have a number of barriers to the adoption AI systems as part of their decision-making process, including the need of setting up measures for cybersecurity and the need to protect the privacy of citizens and their data. Both aspects have implications on human rights regarding the issues of surveillance, tracking, communication, and data storage, as well as automation of processes without rigorous ethical standards^21^. Targeting these gaps would be essential to ensure the usability and practicality of AI technologies for governments. This would also be a prerequisite for understanding long-term impacts of AI regarding its potential, while regulating its use to reduce the possible bias that can be inherent to AI^6^.

Furthermore, our research suggests that AI applications are currently biased towards SDG issues that are mainly relevant to those nations where most AI researchers live and work. For instance, many systems applying AI technologies to agriculture, e.g., to automate harvesting or optimize its timing, are located within wealthy nations. Our literature search resulted in only a handful of examples where AI technologies are applied to SDG-related issues in nations without strong AI research. Moreover, if AI technologies are designed and developed for technologically advanced environments, they have the potential to exacerbate problems in less wealthy nations (e.g., when it comes to food production). This finding leads to a substantial concern that developments in AI technologies could increase inequalities both between and within countries, in ways which counteract the overall purpose of the SDGs. We encourage researchers and funders to focus more on designing and developing AI solutions, which respond to localized problems in less wealthy nations and regions. Projects undertaking such work should ensure that solutions are not simply transferred from technology-intensive nations. Instead, they should be developed based on a deep understanding of the respective region or culture to increase the likelihood of adoption and success.

## Towards sustainable AI

The great wealth that AI-powered technology has the potential to create may go mainly to those already well-off and educated, while job displacement leaves others worse off. Globally, the growing economic importance of AI may result in increased inequalities due to the unevenly distributed educational and computing resources throughout the world. Furthermore, the existing biases in the data used to train AI algorithms may result in the exacerbation of those biases, eventually leading to increased discrimination. Another related problem is the usage of AI to produce computational (commercial, political) propaganda based on big data (also defined as “big nudging”), which is spread through social media by independent AI agents with the goals of manipulating public opinion and producing political polarization^51^. Despite the fact that current scientific evidence refutes technological determinism of such fake news^51^, long-term impacts of AI are possible (although unstudied) due to a lack of robust research methods. A change of paradigm is therefore needed to promote cooperation and to limit the possibilities for control of citizen behavior through AI. The concept of Finance 4.0 has been proposed^52^ as a multi-currency financial system promoting a circular economy, which is aligned with societal goals and values. Informational self-determination (in which the individual takes an active role in how their data are handled by AI systems) would be an essential aspect of such a paradigm^52^. The data intensiveness of AI applications creates another problem: the need for more and more detailed information to improve AI algorithms, which is in conflict with the need of more transparent handling and protection of personal data^53^. One area where this conflict is particularly important is healthcare: Panch et al.^54^ argue that although the vast amount of personal healthcare data could lead to the development of very powerful tools for diagnosis and treatment, the numerous problems associated to data ownership and privacy call for careful policy intervention. This is also an area where more research is needed to assess the possible long-term negative consequences. All the challenges mentioned above culminate in the academic discourse about legal personality of robots^55^, which may lead to alarming narratives of technological totalitarianism.

**Fig. 5:**
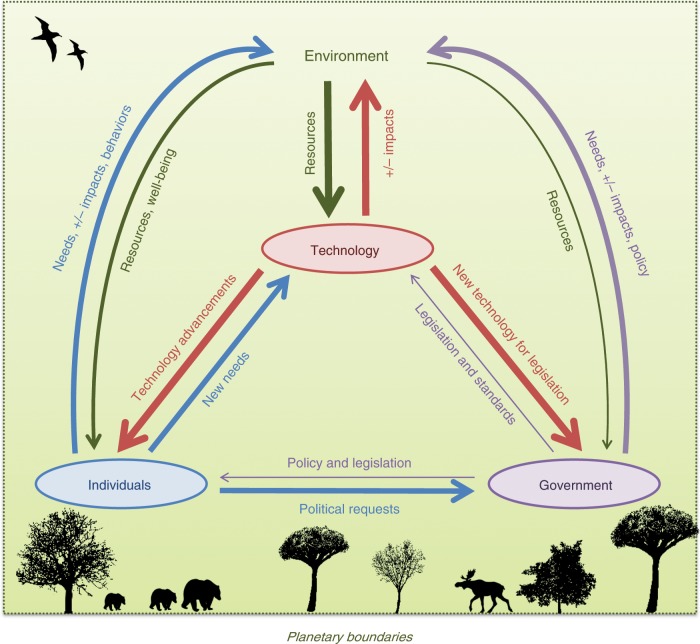
Interaction of AI and society. Schematic representation showing the identified agents and their roles towards the development of AI. Thicker arrows indicate faster change. In this representation, technology affects individuals through technical developments, which change the way people work and interact with each other and with the environment, whereas individuals would interact with technology through new needs to be satisfied. Technology (including technology itself and its developers) affects governments through new developments that need appropriate piloting and testing. Also, technology developers affect government through lobbying and influencing decision makers. Governments provide legislation and standards to technology. The governments affect individuals through policy and legislation, and individuals would require new legislation consistent with the changing circumstances from the governments. The environment interacts with technology by providing the resources needed for technological development and is affected by the environmental impact of technology. Furthermore, the environment is affected either negatively or positively by the needs, impacts, and choices of individuals and governments, which in turn require environmental resources. Finally, the environment is also an underlying layer that provides the “planetary boundaries” to the mentioned interactions.

Many of these aspects result from the interplay between technological developments on one side and requests from individuals, response from governments, as well as environmental resources and dynamics on the other. Figure 5 shows a schematic representation of these dynamics, with emphasis on the role of technology. Based on the evidence discussed above, these interactions are not currently balanced and the advent of AI has exacerbated the process. A wide range of new technologies are being developed very fast, significantly affecting the way individuals live as well as the impacts on the environment, requiring new piloting procedures from governments. The problem is that neither individuals nor governments seem to be able to follow the pace of these technological developments. This fact is illustrated by the lack of appropriate legislation to ensure the long-term viability of these new technologies. We argue that it is essential to reverse this trend. A first step in this direction is to establish adequate policy and legislation frameworks, to help direct the vast potential of AI towards the highest benefit for individuals and the environment, as well as towards the achievement of the SDGs. Regulatory oversight should be preceded by regulatory insight, where policymakers have sufficient understanding of AI challenges to be able to formulate sound policy. Developing such insight is even more urgent than oversight, as policy formulated without understanding is likely to be ineffective at best and counterproductive at worst.

Although strong and connected institutions (covered by SDG 16) are needed to regulate the future of AI, we find that there is limited understanding of the potential impact of AI on institutions. Examples of the positive impacts include AI algorithms aimed at improving fraud detection^56,57^ or assessing the possible effects of certain legislation^58,59^. Another concern is that data-driven approaches for policing may hinder equal access to justice because of algorithm bias, particularly towards minorities^60^. Consequently, we believe that it is imperative to develop legislation regarding transparency and accountability of AI, as well as to decide the ethical standards to which AI-based technology should be subjected to. This debate is being pushed forward by initiatives such as the IEEE (Institute of Electrical and Electronics Engineers) ethical aligned design^60^ and the new EU (European Union) ethical guidelines for trustworthy AI^61^. It is noteworthy that despite the importance of an ethical, responsible, and trustworthy approach to AI development and use, in a sense, this issue is independent of the aims of the article. In other words, one can envision AI applications that improve SDG outcomes while not being fully aligned with AI ethics guidelines. We therefore recommend that AI applications that target SDGs are open and explicit about guiding ethical principles, also by indicating explicitly how they align with the existing guidelines. On the other hand, the lack of interpretability of AI, which is currently one of the challenges of AI research, adds an additional complication to the enforcement of such regulatory actions^62^. Note that this implies that AI algorithms (which are trained with data consisting of previous regulations and decisions) may act as a “mirror” reflecting biases and unfair policy. This presents an opportunity to possibly identify and correct certain errors in the existing procedures. The friction between the uptake of data-driven AI applications and the need of protecting the privacy and security of the individuals is stark. When not properly regulated, the vast amount of data produced by citizens might potentially be used to influence consumer opinion towards a certain product or political cause^51^.

AI applications that have positive societal welfare implications may not always benefit each individual separately^41^. This inherent dilemma of collective vs. individual benefit is relevant in the scope of AI applications but is not one that should be solved by the application of AI itself. This has always been an issue affecting humankind and it cannot be solved in a simple way, since such a solution requires participation of all involved stakeholders. The dynamicity of context and the level of abstraction at which human values are described imply that there is not a single ethical theory that holds all the time in all situations^63^. Consequently, a single set of utilitarian ethical principles with AI would not be recommendable due to the high complexity of our societies^52^. It is also essential to be aware of the potential complexity in the interaction between human and AI agents, and of the increasing need for ethics-driven legislation and certification mechanisms for AI systems. This is true for all AI applications, but especially those that, if they became uncontrolled, could have even catastrophic effects on humanity, such as autonomous weapons. Regarding the latter, associations of AI and robotics experts are already getting together to call for legislation and limitations of their use^64^. Furthermore, associations such as the Future of Life Institute are reviewing and collecting policy actions and shared principles around the world to monitor progress towards sustainable-development-friendly AI^65^. To deal with the ethical dilemmas raised above, it is important that all applications provide openness about the choices and decisions made during design, development, and use, including information about the provenance and governance of the data used for training algorithms, and about whether and how they align with existing AI guidelines. It is therefore important to adopt decentralized AI approaches for a more equitable development of AI^66^.

We are at a critical turning point for the future of AI. A global and science-driven debate to develop shared principles and legislation among nations and cultures is necessary to shape a future in which AI positively contributes to the achievement of all the SDGs. The current choices to develop a sustainable-development-friendly AI by 2030 have the potential to unlock benefits that could go far-beyond the SDGs within our century. All actors in all nations should be represented in this dialogue, to ensure that no one is left behind. On the other hand, postponing or not having such a conversation could result in an unequal and unsustainable AI-fueled future.

## Methods

In this section we describe the process employed to obtain the results described in the present study and shown in the Supplementary Data 1. The goal was to answer the question “Is there published evidence of AI acting as an enabler or an inhibitor for this particular target?” for each of the 169 targets within the 17 SDGs. To this end, we conducted a consensus-based expert elicitation process, informed by previous studies on mapping SDGs interlinkages^8,9^ and following Butler et al.^67^ and Morgan^68^. The authors of this study are academics spanning a wide range of disciplines, including engineering, natural and social sciences, and acted as experts for the elicitation process. The authors performed an expert-driven literature search to support the identified connections between AI and the various targets, where the following sources of information were considered as acceptable evidence: published work on real-world applications (given the quality variation depending on the venue, we ensured that the publications considered in the analysis were of sufficient quality); published evidence on controlled/laboratory scenarios (given the quality variation depending on the venue, we ensured that the publications considered in the analysis were of sufficient quality); reports from accredited organizations (for instance: UN or government bodies); and documented commercial-stage applications. On the other hand, the following sources of information were not considered as acceptable evidence: educated conjectures, real-world applications without peer-reviewed research; media, public beliefs or other sources of information.

The expert elicitation process was conducted as follows: each of the SDGs was assigned to one or more main contributors, and in some cases to several additional contributors as summarized in the Supplementary Data 1 (here the initials correspond to the author names). The main contributors carried out a first literature search for that SDG and then the additional contributors completed the main analysis. One published study on a synergy or a trade-off between a target and AI was considered enough for mapping the interlinkage. However, for nearly all targets several references are provided. After the analysis of a certain SDG was concluded by the contributors, a reviewer was assigned to evaluate the connections and reasoning presented by the contributors. The reviewer was not part of the first analysis and we tried to assign the roles of the main contributor and reviewer to experts with complementary competences for each of the SDGs. The role of the reviewer was to bring up additional points of view and considerations, while critically assessing the analysis. Then, the main contributors and reviewers iteratively discussed to improve the results presented for each of the SDGs until the analysis for all the SDGs was sufficiently refined.

**Fig. 6:**
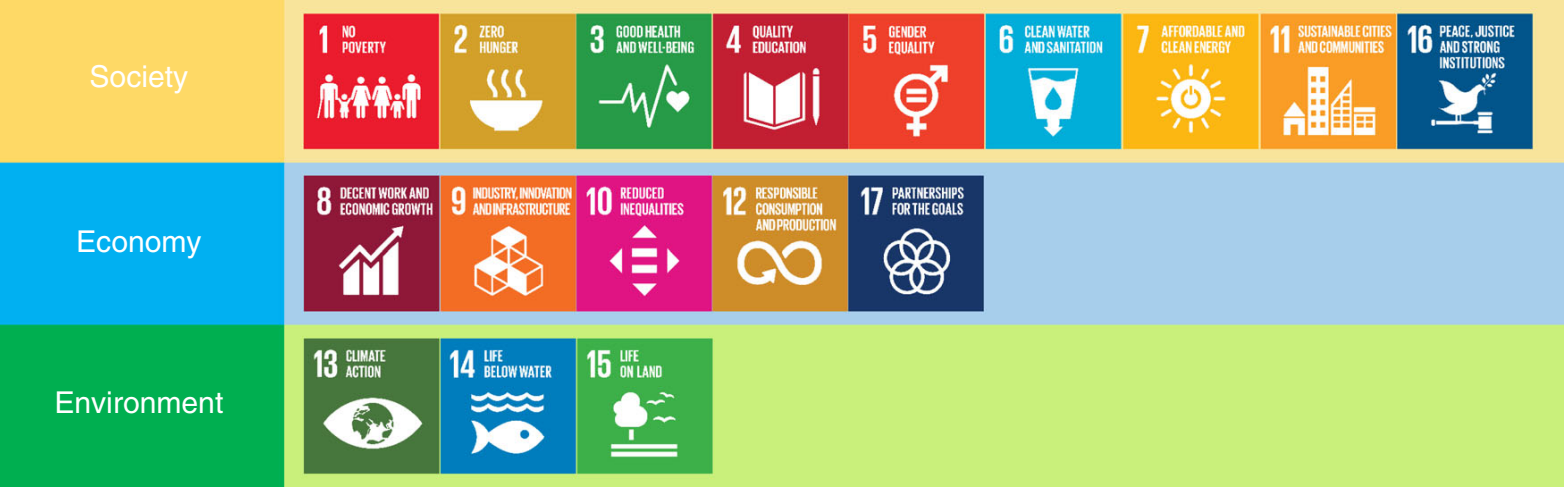
Categorization of the SDGs (https://www.un.org/sustainabledevelopment/) into the Society, Economy, and Environment groups. (The content of this figure has not been reviewed by the United Nations and does not reflect its views).

After reaching consensus regarding the assessment shown in the Supplementary Data 1, we analyzed the results by evaluating the number of targets for which AI may act as an enabler or an inhibitor, and calculated the percentage of targets with positive and negative impact of AI for each of the 17 goals, as shown in Fig. 1. In addition, we divided the SDGs into the three following categories: Society, Economy, and Environment, consistent with the classification discussed by Refs. ^11,12^. The SDGs assigned to each of the categories are shown in Fig. 6 and the individual results from each of these groups can be observed in Figs. 2–4. These figures indicate, for each target within each SDG, whether any published evidence of positive or negative impact was found.

### Taking into account the types of evidence

In the methodology described above, a connection between AI and a certain target is established if at least one reference documenting such a link was found. As the analyzed studies rely on very different types of evidence, it is important to classify the references based on the methods employed to support their conclusions. Therefore, all the references in the Supplementary Data 1 include a classification from (A) to (D) according to the following criteria:

- References using sophisticated tools and data to refer to this particular issue and with the possibility to be generalized are of type (A).
- Studies based on data to refer to this particular issue, but with limited generalizability, are of type (B).
- Anecdotal qualitative studies and methods are of type (C) .
- Purely theoretical or speculative references are of type (D).

The various classes were assigned following the same expert elicitation process described above. Then, the contribution of these references towards the linkages is weighted and categories (A), (B), (C), and (D) are assigned relative weights of 1, 0.75, 0.5, and 0.25, respectively. It is noteworthy that, given the vast range of studies on all the SDG areas, the literature search was not exhaustive and, therefore, certain targets are related to more references than others in our study. To avoid any bias associated to the different amounts of references in the various targets, we considered the largest positive and negative weight to establish the connection with each target. Let us consider the following example: for a certain target, one reference of type (B) documents a positive connection and two references of types (A) and (D) document a negative connection with AI. In this case, the potential positive impact of AI on that target will be assessed with 0.75, while the potential negative impact is 1.

### Limitations of the research

The presented analysis represents the perspective of the authors. Some literature on how AI might affect certain SDGs could have been missed by the authors or there might not be published evidence yet on such interlinkage. Nevertheless, the employed methods tried to minimize the subjectivity of the assessment. How AI might affect the delivery of each SDG was assessed and reviewed by several authors and a number of studies were reviewed for each interlinkage. Furthermore, as discussed in the Methods section, each interlinkage was discussed among a subset of authors until consensus was reached on its nature.

Finally, this study relies on the analysis of the SDGs. The SDGs provide a powerful lens for looking at internationally agreed goals on sustainable development and present a leap forward compared with the Millenium Development Goals in the representation of all spheres of sustainable development, encompassing human rights^69^, social sustainability, environmental outcomes, and economic development. However, the SDGs are a political compromise and might be limited in the representation of some of the complex dynamics and cross-interactions among targets. Therefore, the SDGs have to be considered in conjunction with previous and current, and other international agreements^9^. For instance, as pointed out in a recent work by UN Human Rights^69^, human rights considerations are highly embedded in the SDGs. Nevertheless, the SDGs should be considered as a complement, rather than a replacement, of the United Nations Universal Human Rights Charter^70^.

## Supplementary information

Description of Additional Supplementary Files

Supplementary Data 1

## Data Availability

The authors declare that all the data supporting the findings of this study are available within the paper and its Supplementary Data 1 file.
