# Supplementary material for: The role of artificial intelligence in achieving the Sustainable Development Goals
Source: Nat Commun. 2020 Jan 13;11:233. doi: 10.1038/s41467-019-14108-y (PMC6957485; doi:10.1038/s41467-019-14108-y)
Supplement: Supplementary file 1 — Description of Additional Supplementary Files [file 41467_2019_14108_MOESM1_ESM.pdf]

Description of Additional Supplementary Files

File Name: Supplementary Data 1
Description: Identified positive and negative impacts of AI on each of the SDG targets, including reasoning and references.
